# Clinical and Cognitive Functioning Changes After Partial Hospitalization in Patients With Anorexia Nervosa

**DOI:** 10.3389/fpsyt.2021.653506

**Published:** 2021-04-20

**Authors:** Elena Tenconi, Enrico Collantoni, Valentina Meregalli, Elisa Bonello, Tatiana Zanetti, Angela Veronese, Paolo Meneguzzo, Angela Favaro

**Affiliations:** ^1^Department of Neuroscience, University of Padova, Padua, Italy; ^2^Padova Neuroscience Center, University of Padova, Padua, Italy

**Keywords:** anorexia nervosa, cognitive functioning, executive functions, partial hospitalization, DH treatment, longitudinal assessment, follow-up

## Abstract

**Introduction:** Anorexia nervosa is usually associated with emotional and cognitive difficulties. Little knowledge is available about the changes in cognitive functioning in patients undergoing treatments. The aim of the present study was to longitudinally assess the impact of partial hospitalization on clinical and cognitive functioning in anorexia nervosa.

**Materials and Methods:** 56 women with anorexia nervosa according to DSM-5 criteria and 58 healthy women were enrolled in the study. At baseline, all participants underwent clinical, diagnostic and neuropsychological assessment (T0). Patients were also assessed at the end of the treatment program (T1; *n* = 56).

**Results:** BMI improved significantly throughout treatment. At baseline, patients showed significantly poorer executive abilities and less specific autobiographical memory. After the day-hospital program, decision-making abilities improved significantly. Response to treatment was predicted by BMI at admission and duration of illness, but neuropsychological performance did not contribute to the prediction model.

**Discussion:** Cognitive difficulties, mostly regarding executive functions, resulted differently affected by clinical improvement. In particular, while cognitive monitoring and cognitive inhibition appear to be mostly stable trait-like characteristics, decision-making is both more state-dependent and sensitive to clinical status. None of the cognitive variables added information about the response to day hospital treatment; patients with short duration of illness and a rapidly decreasing BMI would benefit more from intensive interventions than less “acute” patients. These observations, if confirmed by future studies, have important clinical implications in order to understand the impact of malnutrition on cognitive functioning and to provide individualized effective treatment for patients with anorexia nervosa.

## Introduction

Eating disorders are very complex psychiatric manifestations characterized by an over-appraisal of food, eating, body weight and shapes, along with an extreme need for control over these aspects (1). Studies on the effectiveness of psychological treatments for eating disorders, especially for anorexia nervosa, found no superiority of a specific approach (2, 3) and, for adolescent patients, family-based interventions are the dominant model. Following the international clinical guidelines, a higher intensity of care (i.e., day hospital and/or hospitalization) in both adolescents and adults is indicated when there is a high medical risk or a lack of response to outpatient treatment (4). More intensive interventions appear effective in gradually improving nutritional status and controlling dysfunctional behaviors (i.e., binging, purging, hyperactivity, other manifestations of impulsivity), although they are also associated with a high risk of subsequent relapse. Ambivalence and poor motivation are important aspects of the disorder and may be associated with several factors including neurocognitive difficulties, such as cognitive rigidity, difficulties in global thinking, and emotional/social difficulties.

In recent years, based on these clinical observations, researchers have started to systematically assess cognitive functioning in eating disorders, not only in terms of body weight and shape distortions or overvaluation of food and eating, but specifically with regard to the way in which patients think and how they cope with difficulties. This has led research interest to be extended to a broad cognitive and neuropsychological profile of these complex disorders (5, 6). The neuropsychological phenotype of anorexia nervosa consists of cognitive rigidity, set-shifting difficulties, high involvement in rigid and repetitive habits that obstructs the ability to infer environmental changes and the need for new response strategies (leading to perseveration), poor central coherence (i.e., higher detail-focused information processing despite global thinking), poor foresight in decision-making, and a sort of context-dependence in adaptive decision-making, along with difficulties in stopping automatic (hyper-learned) responses in place of more creative and adaptive answers (7–11). Some of these aspects appear to persist after recovery and have been detected, albeit more subtly, also in unaffected sisters (9, 12–14). Nevertheless, some evidence has been provided that the morbid process, in terms of illness duration, illness state and illness severity, negatively impacts cognitive performance (8). Furthermore, there is some evidence which suggests differential neuropsychological alterations underlying the spectrum of eating disorders (restrictive vs. binge-purging extremes), especially with regard to executive functions (in particular, set-shifting and decision-making) (15, 16). The real impact of malnutrition and being underweight on cognitive functions and the effective role of neurocognitive alterations toward clinical expressions and outcome have not yet been defined (5). For these reasons, there is a need to carry out longitudinal studies in order to better clarify these aspects (e.g., their state/trait nature of cognitive alterations in anorexia nervosa), in particular, given the relevance of cognitive abilities as factors involved at different levels and in several aspects (i.e., vulnerability, maintaining factors, treatment motivation and adherence, treatment outcome and prognosis). Moreover, the impact of care not specifically oriented toward cognitive rehabilitation on clinical and cognitive improvement remains to be clarified. Treatments directly targeting cognitive alterations (e.g., cognitive remediation therapy) do not seem to improve clinical outcomes and the data on their efficacy on cognitive functions are mixed (17). The main aim of the present study was to evaluate the longitudinal changes of clinical, psychopathological and cognitive characteristics in a group of patients with anorexia nervosa who were admitted to an intensive semi-residential treatment program as a step of their therapeutic pathway. We also aimed to explore the predictive role of clinical and cognitive characteristics in treatment outcomes.

## Methods

In the first part of the study, we performed a cross-sectional comparison between a group of adolescent and young adult female individuals with anorexia nervosa who completed a partial-hospitalization treatment (T0) and a group of healthy women of similar age and education. Then, we conducted a longitudinal study with the same patients, who were also assessed at the end of the treatment (T1) and, in a small subgroup, at 1-year follow-up (T2). Given that both data collection is still in progress and this subgroup has a small sample size (N 19), we focus analyses on the data pertaining to the first two times (T0 and T1).

### Participants and Treatment

Fifty-six patients with anorexia nervosa, according to the DSM-5 criteria (18), and 58 healthy controls were included in the study. We considered eligible for the study all patients consecutively admitted to the day-hospital (DH) treatment program at the Eating Disorders Unit of the Padova University Hospital (Italy), from July 2014 to July 2020. Inclusion criteria for patients were: admission to our Unit's DH treatment for anorexia nervosa, more than 14 years of age, written informed consent, by the patients or parents (in patients under 18 years of age) for participation in the study. Exclusion criteria were: prior or current traumatic brain injury, lifetime history of any neurological, systemic and/or severe psychiatric illness in comorbidity with AN (suicidality, alcohol/substance use, psychotic features), drop-out from treatment, need for acute admission to a medical ward, admission for bulimia nervosa. The exclusion criteria for controls, recruited from the general population, were body mass index (BMI) below 18, having a first-degree relative with a lifetime eating disorder, prior or current traumatic brain injury, any neurological, psychiatric, or systemic illness and use of psychoactive medication. In the 6 years considered in the study, 122 patients were admitted to the DH. Seventy-one patients were excluded for the following reasons: 12 were admitted with a bulimia nervosa diagnosis, 14 dropped out, 20 were admitted to the DH only to manage acute medical complications or while waiting for admission to a full-day hospital program, three were males, three were pregnant, three were still in treatment, 11 because of other exclusion criteria.

In our ED Unit, admission to day-hospital has to be considered one step of a multi-disciplinary treatment that is indicated: (1) when outpatient treatment is not effective; (2) when patients need urgent assistance in order to stop weight loss; (3) for patients who need intensive support for particular situations (pregnancy, waiting for admission to a full-day intensive program). The DH-treatment program is a 5-day per week treatment offered between Monday and Friday from 9:30 am to 4:30 pm. The duration of the treatment is flexible and tailored on the specific needs of patients. The program is cognitive-behaviorally oriented, and during the DH treatment each patient has both individualized and group psychotherapy sessions. The nutritional program is also individualized and discussed with an expert dietician who follows the patient throughout the course of the treatment. The main activities in DH are assisted meals, “Monday clinical meetings” of the patient with the psychotherapist and the dietician, individual psychotherapeutic sessions twice a week and, usually, group psychotherapy once a week; a group about the “rules” of the day-hospital program is also conducted every 2 weeks; psychoeducation and other activities, such as relaxation training, are also included in the program. Patients are regularly monitored by an expert internal medicine physician during the whole treatment.

All participants to the study were assessed by means of a clinical, psychopathological and neuropsychological test battery. Participants were weighed and measured in height, and underwent an adapted version of the eating disorders section of the Structured clinical interview for DSM-5 (19), and a semi-structured interview to collect sociodemographic and clinical information. The neuropsychological assessment was administered during the morning in a quiet and comfortable room in a 90 min session. Computerized tasks were administered using a 15^′′^ notebook and test presentation was counterbalanced across all participants. For patients, data were collected at DH admission (T0, within the 1st week of admission) and at discharge (T1). The Edinburgh Handedness Inventory (20) was administered to assess hand lateralization. The study was approved by the Ethical Committee of the Padova University Hospital.

Improvement was based on both BMI normalization and the clinical judgement of the two therapists who evaluated and followed the patients during the day-hospital treatment. In particular, we considered the following items as a sign of improvement, if present together: (1) an increase in BMI by at least three percentile points; (2) a general improvement in eating patterns in terms of pace, amount, and variety of foods accepted and regularly taken; (3) a significant modification in global functioning (i.e., restoration of school/work attendance, if previously interrupted, resumption of social relationships and exchanges); (4) a reduction in depressive and anxious symptoms as detected by self-reported assessment, but also as reported by both the patient him/herself and significant others.

### Self-Reported Measures

The State Trait-Anxiety Inventory (STAI) (21) was administered only at T0 to assess state and trait anxiety, while the following questionnaires were administered at both T0 and T1: the Hopkins Symptom Checklist (HSCL-58) (22) to assess psychiatric symptoms, the Eating Disorder Inventory (EDI) (23) to detect eating psychopathology, and the Body Attitude Test (BAT) (24) to investigate body image experience of one's body.

### Cognitive Assessment

A broad neuropsychological assessment (9, 11) covering several cognitive settings was administered to both patients and controls.

The tasks used only at T0 were:

- the Brief Intelligence Test (TIB) (25), the Italian version of the National Adult Reading Test, to measure premorbid general cognitive abilities;
- the Wisconsin Card Sorting Task (WCST) (26, 27), which is a widely used abstract thinking, set-shifting and cognitive flexibility task (9);
- the Rey-Osterrieth Complex Figure Test (ROCFT) (28) is a task involving both perceptive and executive (planning) abilities (9).

The tasks administered at all the assessments were the following:

- the Iowa Gambling Task (IGT) (29), which is a well-known and previously described computer task investigating decision-making under risk (11, 30);
- the Cognitive Bias Task (CBias) (31), which measures adaptive decision-making in terms of a balance between context-independent (based on preexisting internal representations) and context-dependent (based on the current features characterizing that specific scenario) decision-making (see previous descriptions of the task in 11);
- the Stop-Signal Task (SST) (32), which is a paradigm developed to measure cognitive monitoring and response inhibition (10);
- the Mittenecker Pointing Test (MPT) (33), which is a random-motor-generation task examining two components of cognitive flexibility (inhibition of prepotent responses and memory monitoring/updating). Participants are instructed to press nine unlabeled keys irregularly distributed over the computer board following an acoustic signal (1.2/s) which monitors the response production rate (i.e., one key pressed for each sound). The task demands pressing keys in the most random order possible and is in contrast with our innate tendency to produce repetitive sequences, so a continuously high control effort is required to inhibit automatically developing routines. The total responses are 180 and the outcome variables are two quantitative measures of deviation from randomness: Context Redundancy (CR) and Symbol Redundancy (SR). CR assesses the inhibition of developing routines (response sequences) and SR gives a measure of the memory component (memory monitoring/updating) of random sequence generation. Both values range from 0 to 1. Low CR and SR values are associated with high cognitive flexibility.
- the Autobiographical Memory Task (AMT) (34), which assesses the ability to retrieve specific episodes from one's own life, by means of cue-words varying in their emotional valence (i.e., positive, negative, neutral). The task consisted in orally presenting 12 cue-words and participants were instructed to recall a specific episodic memory (with a limited space-time location) from their life, that had happened more than a week before (not a current episode). Outcome measures considered were: the total specific memories retrieved, over-general memories (categorical + extended memories), recent memories (specific episodes pertaining to the last 3 months), omissions (failure to respond in 30”), time to complete the task and the emotional valence of memories retrieved regardless of cue-word valence.

### Practice Effect

In order to reduce the risk of a learning effect of the task, which should always be considered in planning a longitudinal study (35), we adopted, where possible, alternative versions of the same task. In particular, for the IGT, we changed the output of the different decks (with regard to the magnitude and the frequency of losses). For the AMT, we adopted three alternate versions of the task, two proposed by Williams (34) and the third derived from cues adopted by other autobiographical memory tasks proposed by the same group of researchers. For the CBias, the SST and the MPT we did not expect any practice effect. In the SST, the outputs are in terms of reaction times and parameters (i.e., the frequency and time of the stop-signal) change stochastically. The CBias and the MPT are covert tasks, where a participant does not know exactly what he/she is doing, and it is difficult for participants to learn anything or understand the rationale of the task.

### Statistical Analyses

Non-parametrical statistics were used to compare not normally distributed groups. In particular, the U Mann-Whitney test was used for non-parametric comparisons between two independent groups. The Wilcoxon rank sum test for two paired groups was employed to test longitudinally data. Associations between variables were tested by means of the Spearman Rho. The General Linear Model with age as covariate was used to compare more than two groups. Multivariate logistic regression models were used to assess the predictive role of clinical, psychopathological and neuropsychological variables on clinical outcome. In order to control the multi-comparison bias, the Bonferroni correction was used, and only *p*-values equal to or lower than 0.003 were considered significant. All statistical procedures were conducted by means the SPSS, version 26 (IBM, 2019).

## Results

### Treatment Outcome

In the whole sample (*n* = 56), the average duration of the day-hospital program was 23.0 weeks (SD = 11.3), corresponding to 78.0 days of treatment (SD = 39.1). All patients completed assessment at T0 and T1. Most patients belonged to the restrictive subtype (*n* = 48; 86%) and 20 were taking antidepressant drugs at the time of admission (36%). The average BMI was 15.6 (SD = 1.5; range 12.4–18.2) at admission and 17.3 (SD = 1.7; range 14.5–22.7) at discharge.

Out of the 56 patients enrolled in the study, 16 (29%) showed no improvement during the program. Patients (*n* = 40) who improved during the partial hospitalization program had a BMI of 15.3 (SD = 1.5; range 12.4–18.2) at admission and 17.6 (SD = 1.8) at discharge. All patients who did not improve during the day-hospital program were referred for more intensive treatment. Out of those patients who improved during the day-hospital program, four decided to undergo a full-day intensive treatment and the others to complete their treatment in an outpatient setting.

### Patients vs. Healthy Women

Table 1 summarizes the clinical and general characteristics of patients with anorexia nervosa and healthy women, including hand lateralization, depressive symptoms and state/trait anxiety. Patients with AN showed significantly higher scores (*p* < 0.001) than healthy women on all the subscales of the EDI and the H-SCL.

**Table 1:** Clinical and general characteristics of patients (at admission) and controls.

	**Anorexia nervosa (*n* = 56) Mean (SD)**	**Healthy women (*n* = 58) Mean (SD)**	***Z***	***p***
Age (years)	19.6 (5.2)	19.5 (4.9)	−0.14	ns
Education (years)	12.0 (2.3)	11.9 (2.3)	−0.31	ns
BMI (kg/h^2^)	15.6 (1.5)	21.3 (2.2)	−9.03	0.000
Minimum BMI	15.5 (1.6)	19.6 (2.1)	−8.20	0.000
Age of onset (years)	16.4 (3.6)	—		
Illness duration (months)	23.9 (26.0)	—		
N° previous treatments	1.02 (1.23)	—		
Hand lateralization (Edinburgh score)	57.1 (29.9)	66.8 (25.1)	−1.87	0.061
Trait anxiety (STAI)	56.1 (13.4)	41.4 (9.6)	−6.045	0.000
State anxiety (STAI)	48.5 (13.2)	34.7 (7.1)	−5.894	0.000
Depression (H-SCL)	1.82 (0.9)	0.68 (0.6)	−5.566	0.000

*STAI, State Trait Anxiety Inventory; H-SCL, Hopkins Symptoms Checklist*.

Patients and controls did not differ in estimated intelligence quotients investigated by means of the Brief Intelligence Test (103.6 ± 4.2 vs. 105.5 ± 4.3; *z* = 1.77; *p* = 0.083). Table 2 reports the comparison between patients and controls in the neuropsychological tasks.

**Table 2:** Neuropsychological tasks in patients with AN and healthy controls.

	**Anorexia nervosa (*n* = 56) Mean (SD)**	**Healthy women (*n* = 58) Mean (SD)**	ANOVA^*^F_(1,112)_	***p***
WCST Global score	51.0 (40.1)	37.8 (27.5)	3.94	0.050
WCST number of categories	4.8 (1.9)	5.8 (1.0)	10.20	0.002
WCST correct answers	66.5 (9.9)	70.6 (8.2)	5.47	0.021
WCST perseverative responses	18.9 (17.0)	12.8 (9.7)	5.08	0.026
IOWA net score	2.0 (30.8)	13.4 (20.8)	5.79	0.018
CBias raw scores	173.6 (29.3)	180.4 (31.9)	1.66	0.200
CBias converted scores	30.0 (22.6)	37.6 (22.7)	3.51	0.064
Rey copy trial	27.9 (3.1)	28.6 (3.8)	1.26	0.264
Rey copi CCI	1.01 (0.45)	1.03 (0.42)	0.12	0.730
Rey memory trial	19.0 (5.3)	19.1 (4.5)	0.003	0.953
Rey total time	201.5 (87.2)	159.1 (45.8)	9.95	0.002
Stop-signal reaction time	243.2 (45.9)	253.5 (46.3)	1.22	0.272
MPT_CR	0.28 (0.17)	0.26 (0.14)	0.16	0.691
MPT_SR	0.03 (0.05)	0.03 (0.08)	0.04	0.847
MPT_Lateral preference	−0.08	−0.1	0.47	0.490
AMT total score	8.9 (2.1)	9.9 (2.2)	5.62	0.020

* *Age was included as a covariate. According to the Bonferroni correction p should be considered at <0.003*.

*WCST, Wisconsin Card Sorting Task; CBias, Cognitive Bias Task; Rey copy CCI, Rey copy Central Coherence Index; MPT_CR, Mittenecker Pointing Task Context Redundancy; MPT_SR, Mittenecker Pointing Task Symbol Redundancy; AMT total score, Autobiographical Memory Task total specific memories*.

The patients of our study showed altered executive functioning (Table 2) and trends for difficulties in decision-making, autobiographical memory, and they took longer to complete the direct copy of the ROCFT. On the AMT patients showed a poorer ability to retrieve their own life episodes, needed more time to retrieve memories (*F* = 5.12; *p* = 0.026) and reported a higher number of general memories than specific autobiographical episodes (*F* = 6.87; *p* = 0.010), compared to healthy controls. Moreover, in response to positive cues patients reported significantly fewer memories (*F* = 11.03; *p* = 0.001) than controls.

The comparison between patients of the restrictive subtype (*n* = 48) and those of the binge eating/purging type (*n* = 8) showed differences on both the number of perseverative responses (20.9 ± 17.5 vs. 6.7 ± 2.5; *F* = 5.00; *p* = 0.03) and the global score (56.1 ± 40.8 vs. 21.4 ± 16.9; *F* = 5.38; *p* = 0.024). Restrictive patients were also both significantly more underweight at baseline (BMI: 15.4 ± 1.5 vs. 16.9 ± 1.2; *F* = 7.89; *p* = 0.007) and less depressed (1.67 ± 0.89 vs. 2.66 ± 0.84; *F* = 8.34; *p* = 0.006).

Table 3 shows baseline characteristics of patients with good and poor treatment outcome. Patients who improved during the day-hospital program did not differ from those who did not with regard to neuropsychological performance at T0 (only a trend for reduced visual memory at the ROCFT was observed in the non-improved group: 16.6 ± 4.1 vs. 20.0 ± 5.5; *F* = 4.44; *p* = 0.040). However, the improved group was more underweight at baseline (BMI: 15.3 ± 1.5 vs. 16.4 ± 1.4; *F* = 6.03; *p* = 0.017) and showed a trend for a shorter duration of illness (19.4 ± 19.1 vs. 36.7 ± 38.6 months; *z* = 1.97; *p* = 0.049).

**Table 3:** Clinical and general characteristics of patients with good and poor outcome at admission.

	**Anorexia nervosa Good outcome (*n* = 40) Mean (SD)**	**Anorexia nervosa Poor outcome (*n* = 16) Mean (SD)**	***Z***	***p***
Age (years)	19.1 (4.6)	20.7 (6.4)	−0.96	ns
Education (years)	12.0 (2.2)	12.1 (2.5)	−0.92	ns
BMI (kg/h^2^)	15.3 (1.5)	16.4 (1.4)	−2.33	0.020
Minimum BMI	15.4 (1.7)	15.9 (1.4)	−1.19	ns
Age of onset (years)	16.5 (4.0)	16.0 (2.2)	−0.14	ns
Illness duration (months)	13.4 (14.1)	20.6 (32.5)	−0.32	ns
N° of previous treatments	1.1 (1.2)	0.7 (1.2)	−1.50	ns
Menarche	12.1 (1.0)	12.3 (1.4)	−0.33	ns
Age at first diet	14.6 (2.0)	14.4 (1.5)	−0.95	ns
Hand lateralization (Edinburgh score)	56.5 (29.3)	58.5 (32.2)	−0.16	ns
Trait anxiety (STAI)	55.9 (11.7)	59.9 (8.7)	−0.96	ns
State anxiety (STAI)	48.0 (13.7)	49.3 (11.9)	−0.55	ns
Depression (H-SCL)	1.7 (0.97)	2.13 (0.86)	−1.35	ns
	**Frequencies**	**Frequencies**	χ^2^	***p***
AN restrictive subtype	34/40 (85%)	14/16 (87,5%)	0.58	ns

### Longitudinal Changes

Table 4 summarizes clinical (BMI) and psychopathological changes in patients at admission and at discharge. At discharge, patients showed a significative improvement in BMI and in the scores of self-reported questionnaires. The improvement in BMI significantly correlated with the duration of the day-hospital treatment both in terms of days spent in day-hospital (rho = 0.56; *p* < 0.001) and in terms of total time in treatment (rho = 0.36; *p* = 0.006).

**Table 4:** Clinical and psychopathological variables across two longitudinal assessment time points.

	**T0 At admission (*n* = 56) Mean (SD)**	**T1At discharge (*n* = 56) Mean (SD)**	**Wilcoxon *Z***	***p***
BMI	15.6 (1.5)	17.3 (1.7)	5.93	<0.0001
Drive for thinness (EDI)	13.2 (7.3)	7.8 (7.6)	3.80	<0.0001
Body attitudes (BAT)	56.4 (15.7)	48.1 (23.9)	2.18	0.029
H-SCL total score	1.20 (0.49)	0.84 (0.54)	3.15	0.002
H-SCL depression	2.19 (0.81)	1.55 (1.03)	3.47	0.001
IOWA net score	2.3 (29.4)	19.1 (33.8)	3.41	0.001
CBias raw scores	174.7 (29.5)	181.7 (30.4)	2.58	0.010
CBias converted scores	31.0 (22.6)	37.5 (22.7)	1.65	ns
Stop-signal reaction time	243.2 (45.9)	250.7 (95.4)	0.73	ns
MPT_CR	0.28 (0.18)	0.26 (0.14)	0.73	ns
MPT_SR	0.03 (0.06)	0.03 (0.02)	0.59	ns
MPT_Lateral preference	−0.08	−0.08	−1.1	ns
AMT total score	8.9 (2.1)	8.5 (2.3)	0.89	ns
AMT Recent memories	2.4 (1.8)	3.2 (2.6)	1.93	0.054

*According to the Bonferroni correction, p should be considered at <0.003.

*EDI, Eating Disorder Inventory; BAT, Body Attitude Test; H-SCL, Hopkins Syptoms Checklist; CBias, Cognitive Bias Task; MPT_CR, Mittenecker Pointing Task Context Redundancy; MPT_SR, Mittenecker Pointing Task Symbol Redundancy; AMT, Autobiographical Memory Task*.

**Figure 1:**
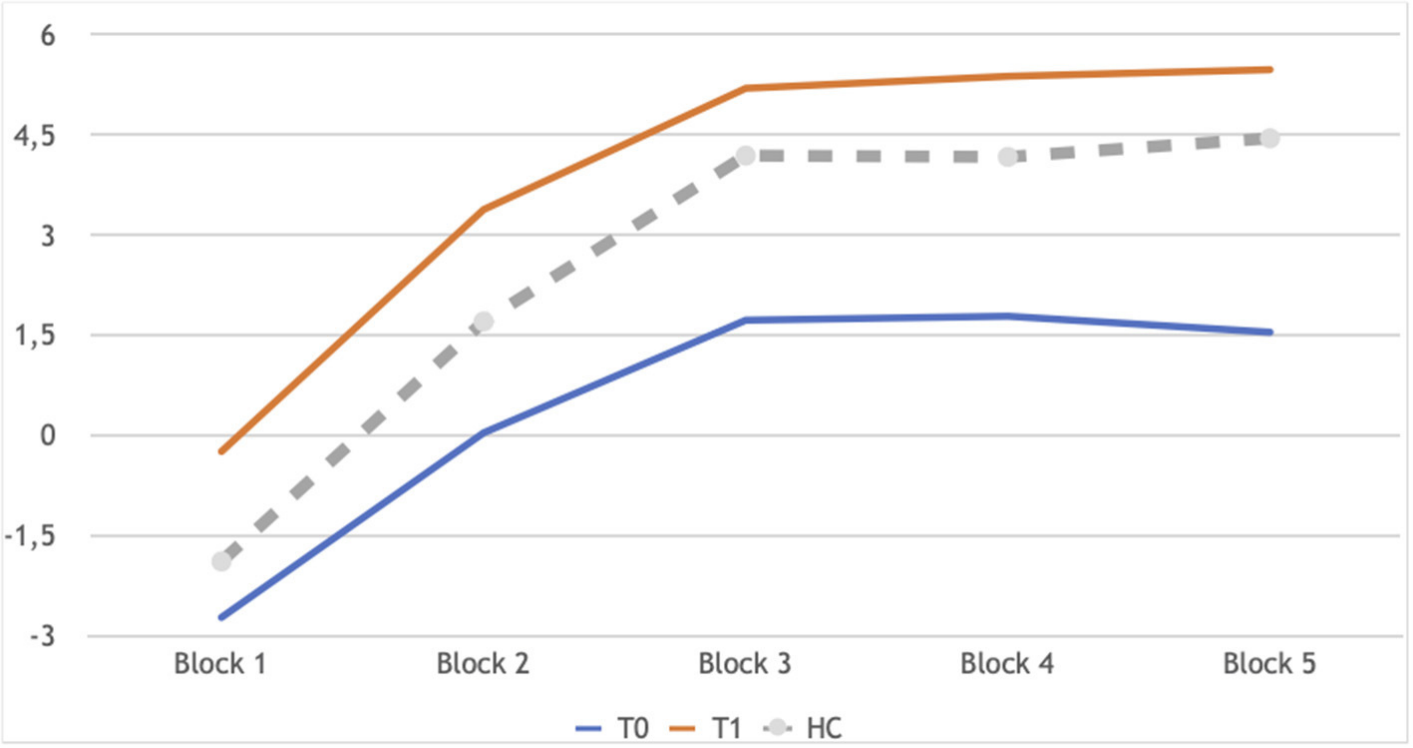
IGT learning profile of patients in longitudinal assessment: T0 and T1 in comparison. Block 1: Z −2.44, *p* 0.014; Block 2:Z −2.73, *p* 0.006; Block 3: Z −2.58, *p* 0.010; Block 4: Z −2.86, *p* 0.004; Block 5: Z −2.68, *p* 0.007. In gray the performance of controls at T0, as a reference, not included in the analyses reported here.

Neuropsychological performance showed few changes between the two time points: a significant improvement in the IGT (Figure 1) and slightly higher CBias raw scores (Table 4).

Using a multivariate logistic regression model to predict the negative outcome at discharge from the DH program we found the following predictors (Model chi-square = 15.41; d.f. = 4; *p* = 0.004; 77% of correct prediction): BMI at admission (T = 0.69; ES = 0.27; Wald = 6.31; *p* = 0.012; OR = 2.00, 95% CI, 1.16–3.43), duration of illness (T = 0.03; ES = 0.01; Wald = 4.15; *p* = 0.042; OR = 1.03, 95% CI, 1.01–1.05), diagnostic subtype (T = 1.90; ES = 1.11; Wald = 2.91; *p* = 0.088; OR = 6.69, 95% CI, 0.75–59.36), and depressive symptoms (T = 0.80; ES = 0.43; Wald = 3.41; *p* = 0.065; OR = 2.22, 95% CI, 0.95–5.18). The inclusion in the model of any other clinical and neuropsychological variable did not improve the predictive ability.

## Discussion

The current longitudinal study complements and strengthens findings from previous studies on the impact of clinical changes on cognitive functioning in anorexia nervosa. It has been hypothesized that cognitive difficulties, especially regarding executive functions and visuospatial information processing, might contribute to maintaining the disorder, both by hindering therapeutic thinking and by reducing illness awareness (36, 37). However, the literature on cognitive changes after clinical improvement is highly conflicting (5). Conspicuous evidence supports the association between anorexia nervosa and executive function alterations. One of the main conceptualizations of executive functions supports their division into three separate key sub-functions moderately related to each other: cognitive shifting, information updating and monitoring and the ability to inhibit automatic responses (38). In our sample we investigated all of these three aspects, firstly cross-sectionally, comparing patients to a group of healthy controls, and then longitudinally, comparing cognitive performances at admission to a DH treatment program and at the end of this program. According to the literature on cross-sectional studies (39), our patients showed poor task-switching and abstract thinking abilities, indicative of cognitive rigidity compared to controls. Differently from some studies (40), but in line with others (39) we observed a greater executive impairment in restrictive patients, compared to those with binge eating/purging symptoms. Updating refers to the ability to check incoming information and to regulate the load of working memory according to the current behavioral goals. This specific aspect has been scarcely investigated in the cognitive research of eating disorders. To our knowledge, this is the first study to apply the MPT, a motor random generation task based on responses which are neither hyper-learned nor linked to academic skills (i.e., counting or spelling), to the anorexia nervosa population. Patients and controls showed very close scores at both indexes (i.e., context and symbol redundancy) and we did not observe specific changes in longitudinal assessments indicating a certain stability and independence from clinical status. One possible reason may be that the CR index taps not so much high-order repetitive behaviors (i.e., compulsions), and narrow interests (the narrowness of focus, inflexibility and perseveration in interests and activities) as low-order repetitive behaviors (simple repetitive motor behaviors) (41). In both cross-sectional and longitudinal assessments patients showed the same pattern exhibited by controls (i.e., higher preference for the right hemispace in both groups) and no differences across time. Finally, we assessed the third sub-executive function, the ability to inhibit or ignore the automatic or dominant tendency to produce a specific (usually hyper-learned) response by the Stop-signal paradigm. Patients investigated here showed no significative differences in SSRT compared to controls. In the longitudinal study, cognitive monitoring and response inhibition did not show an early (T1) appreciable change, indicating a certain independence from the clinical and nutritional status. It may be that BMI normalization is not sufficient to impact response inhibition abilities. The literature on the Stop-signal task in anorexia nervosa lacks longitudinal assessments, and the few cross-sectional neuroimaging studies led to quite mixed results (42). Moreover, one study carried out on recovered anorexia nervosa women (43) and another on adolescent acute anorexia nervosa patients (44) both reported differences in brain activation that were not supported by task performance. In the literature, the few longitudinal studies about decision-making in anorexia found discordant results. In one study patients improved their IGT performance after weight gain especially at one-year follow-up and in the case of complete remission (45), while in the other two studies patients did not improve at all (46, 47). Both the IGT and the CBias in our study showed improvements after treatment, demonstrating greater foresight in making choices (IGT) and greater balance between internal information and context features in adaptive decision-making conditions (CBias). These two tasks assess different kinds of decision-making and our data are compatible with the hypothesis that veridical decision-making abilities are more strictly dependent on physiological and nutritional status, while the adoption of a more adaptive decision-making style appears somewhat related to a more general (both clinically and psychologically) state of well-being. Furthermore, veridical decision-making abilities appear to differentiate patients from controls to a greater extent than adaptive decision-making ability does.

Autobiographical memory, closely linked to superior executive functions, consists in crucial personal memory representations, which set the content of the self and define not only who we are, but also who we have been and who we will become (48). In anorexia nervosa, it is not so rare to clinically observe that, despite the great benefits gained by patients after recovery from the illness, in the case of relapse they have strong difficulty remembering these benefits. Our findings are closely in line with the literature: AN patients, in comparison to controls, retrieved fewer specific memories and more “general” autobiographical ones, a phenomenon called “overgeneral autobiographical memory” (OGM) (49). The hypothesis proposed by the literature is that these general memories have a protective function, allowing patients to diminish the affective impact of life experiences, reducing affective involvement and distress. The Autobiographical Memory Task is considered “a bridge task” between cold and hot cognition. Our patients took longer to complete the task (showing once again a slowness in performing cognitive and emotional tasks) and reported fewer specific memories in response to positive cue-words compared to controls, in line with some literature data which reported a general impairment in access to emotional memories (both positive and negative) (50–52). In the longitudinal assessment, AMT performance appeared to be quite stable, with a trend for a higher number of specific recent episodes (i.e., episodes referring to the last 3 months) at discharge. To our knowledge, this is the first longitudinal study on the AMT in anorexia nervosa.

Concerning the clinical outcome, we found that patients who improved during the DH treatment showed significantly lower BMI along with shorter illness duration. Though it is well-known that a short duration of illness represents a predictor of a positive outcome, the finding about BMI was somewhat unexpected, although not new (53). In the literature about long-term outcome of patients with anorexia nervosa, a lower BMI (or nadir BMI) usually represents a negative predictive factor (54, 55). However, our sample was made up of patients for whom intensive treatment has been indicated and it is possible that our data simply reflect the fact that for more “acute” patients (in terms of both shorter duration of illness and lower BMI) the DH treatment might be more effective and appropriate. A prompt treatment of anorexia nervosa cases is recommended in the literature (56) and early stages of the disorder probably represent a “critical window” within which to act to increase treatment efficacy (57). Our data support the idea that patients with short duration of illness and a rapidly decreasing BMI would benefit more from intensive interventions than less “acute” patients. With the exception of BMI, our findings emerged using a multivariate analysis, with response to treatment as the dependent variable, are in line with the literature, according to which longer duration of illness, the presence of depression, higher age of onset, lower nadir BMI, the presence of bulimic symptoms, and a longer need for in-patient treatment, all are factors associated with a worse outcome (54, 58). We should carefully take into consideration these clinical factors during the diagnostic evaluation, in order to plan individualized and more effective therapeutic projects. It might be the case to recommend partial hospitalization even in patients with short duration of illness and in the presence of low BMI, considering however the importance of depressive and binge eating/purging symptoms as possible barriers to care and potential indications for a full-day intensive approach.

Interestingly, none of the cognitive variables seems to add information about the response to treatment in a partial hospitalization setting. It is possible that an intensive treatment might reduce the importance of cognitive difficulties as possible barriers for an effective treatment. Studies considering other clinical settings should be conducted to better explore this issue. It is also possible that the importance of some cognitive difficulties might be “masked” by those clinical variables that have an impact on cognitive functioning (i.e., decision making). Studies on larger samples are needed to address this point and to confirm our observation of an improvement of some cognitive functions along with an improvement of clinical and nutritional status.

## Conclusions

In summary, the current longitudinal study provides further evidence regarding the presence of cognitive difficulties in patients with anorexia nervosa in their acute stage, with some difficulties persisting despite clinical and nutritional improvement. Executive functioning and autobiographical memory alterations tend to persist beyond clinical recovery and their role as possible vulnerability and maintaining factors needs to be better understood. Decision-making abilities, both veridical and adaptive, were both more state-dependent and sensitive to clinical status changes: their alterations may act as an exacerbation factor in very acute patients.

All these observations, if confirmed by future studies, have important clinical and scientific implications in order to understand the impact of malnutrition on cognitive functioning and to provide individualized effective treatment for patients with anorexia nervosa.

## Data Availability Statement

The raw data supporting the conclusions of this article will be made available by the authors, without undue reservation.

## Ethics Statement

The studies involving human participants were reviewed and approved by Ethical Committee of the Padova University Hospital.

## Author Contributions

ET collected, analyzed the data and wrote the paper. EC analyzed the data and wrote the paper. VM, PM, EB, TZ, and AV, collected data and wrote the paper. AF analyzed data and supervised all steps of the project. ET and EC are the principal investigators of the research, developed the study protocol and supervised the team. All authors contributed to the article and approved the submission version.

## Conflict of Interest

The authors declare that the research was conducted in the absence of any commercial or financial relationships that could be construed as a potential conflict of interest.
